# Type-I Interferons in Alzheimer's Disease and Other Tauopathies

**DOI:** 10.3389/fncel.2022.949340

**Published:** 2022-07-15

**Authors:** Sophie A. I. Sanford, William A. McEwan

**Affiliations:** ^1^Department of Clinical Neurosciences at the University of Cambridge, Cambridge, United Kingdom; ^2^UK Dementia Research Institute at the University of Cambridge, Cambridge, United Kingdom

**Keywords:** type-I interferon response, Alzheimer's disease, tauopathies, innate immunity, antiviral immunity, IFN, amyloid-beta pathology, tau pathology

## Abstract

The detection of pathogen-associated molecular patterns can elicit the production of type-I interferons (IFNs), soluble cytokines that induce a transcriptional state inhibitory to viral replication. Signatures of type-I IFN-driven gene expression, and type-I IFNs themselves, are observed in the central nervous system during neurodegenerative diseases including Alzheimer's disease and other tauopathies, the umbrella term for diseases that feature aggregation of the cytosolic protein tau. The contribution of the type-I IFN response to pathological progression of these diseases, however, is not well-understood. The wholesale transcriptional changes that ensue from type-I IFN production can both promote protective effects and lead to damage dependent on the context and duration of the response. The type-I IFN system therefore represents a signaling pathway with a potential disease-modifying role in the progression of neurodegenerative disease. In this review we summarize the evidence for a type-I IFN signature in AD and other tauopathies and examine the role of aggregated proteins as inflammatory stimuli. We explore both the protective role of IFN against protein pathologies as well as their downstream toxic consequences, which include the exacerbation of protein pathology as a potentially destructive feed-forward loop. Given the involvement of type-I IFNs in other neurogenerative diseases, we draw comparisons with other categories of homotypic protein aggregation. Understanding how type-I IFN influences progression of AD and other tauopathies may yield important insight to neurodegeneration and identify new targets in an area currently lacking disease-modifying therapies.

## Introduction

Alzheimer's disease (AD) is the most common form of dementia and is anticipated to affect more than 113 million people worldwide by 2050 (Knopman et al., 2021). AD is characterized by two distinct pathologies in the post-mortem brain (Stelzmann et al., 1995). Plaques of beta-amyloid (Aβ) peptide, a cleavage product of the transmembrane protein, amyloid precursor protein (APP), accumulate in the extracellular spaces of the brain. In addition, fibrillar and hyperphosphorylated assemblies of the microtubule-associated protein tau accumulate in the cytoplasm of neurons (Goedert and Spillantini, 2006). Mutations in *APP* can lead to dominantly inherited, early-onset variants of AD, though these inherited forms make up <1% of AD cases (Laurent et al., 2018). The “amyloid cascade hypothesis” places Aβ pathology as an upstream, causative insult that unleashes a range of ensuing consequences including tau pathology and neurotoxicity (Hardy and Higgins, 1992). However, clinically targeting Aβ has so far failed to yield cognitive benefit (Karran and De Strooper, 2022). This has directed focus toward other targets such as tau lesions, which correlate strongly with cognitive decline (Nelson et al., 2007).

Tau assemblies are present in a range of neurodegenerative diseases alongside AD, classed as tauopathies. Several non-synonymous point mutations in *MAPT*, the gene that encodes tau, give rise to familial inherited tauopathies such as frontotemporal lobar degeneration with tau-immunoreactive inclusions (FTLD-tau) (Goedert, 2018). These findings establish tau as a causative factor in pathological progression, at least in these rare diseases and potentially more broadly in the tauopathies. In Pick's disease (PiD), progressive supranuclear palsy (PSP) and corticobasal degeneration (CBD), tau pathology is the main, often sole observed protein pathology (Spillantini and Goedert, 2013). The mechanism that leads to the assembly of tau during neurodegeneration is uncertain. Cell-autonomous mechanisms likely drive the earliest tau pathology, which is apparent in the majority of human brains by the age of 30 years (Braak and Del Tredici, 2015). Misfolded tau may also act in a “prion-like” manner promoting its propagation through iterative rounds of seeded aggregation. Injection of mouse brains with tau assemblies can induce tau aggregation in the neurons of the recipient animal (Clavaguera et al., 2009; De Calignon et al., 2012; Liu et al., 2012a). Similar results can also be obtained in cell-based and *ex vivo* models (Frost et al., 2009; Guo and Lee, 2011; McEwan et al., 2017; Miller et al., 2021). While the contribution of this process to disease progression remains undetermined, a unifying feature of AD and several tauopathies is inflammation.

**Figure 1:**
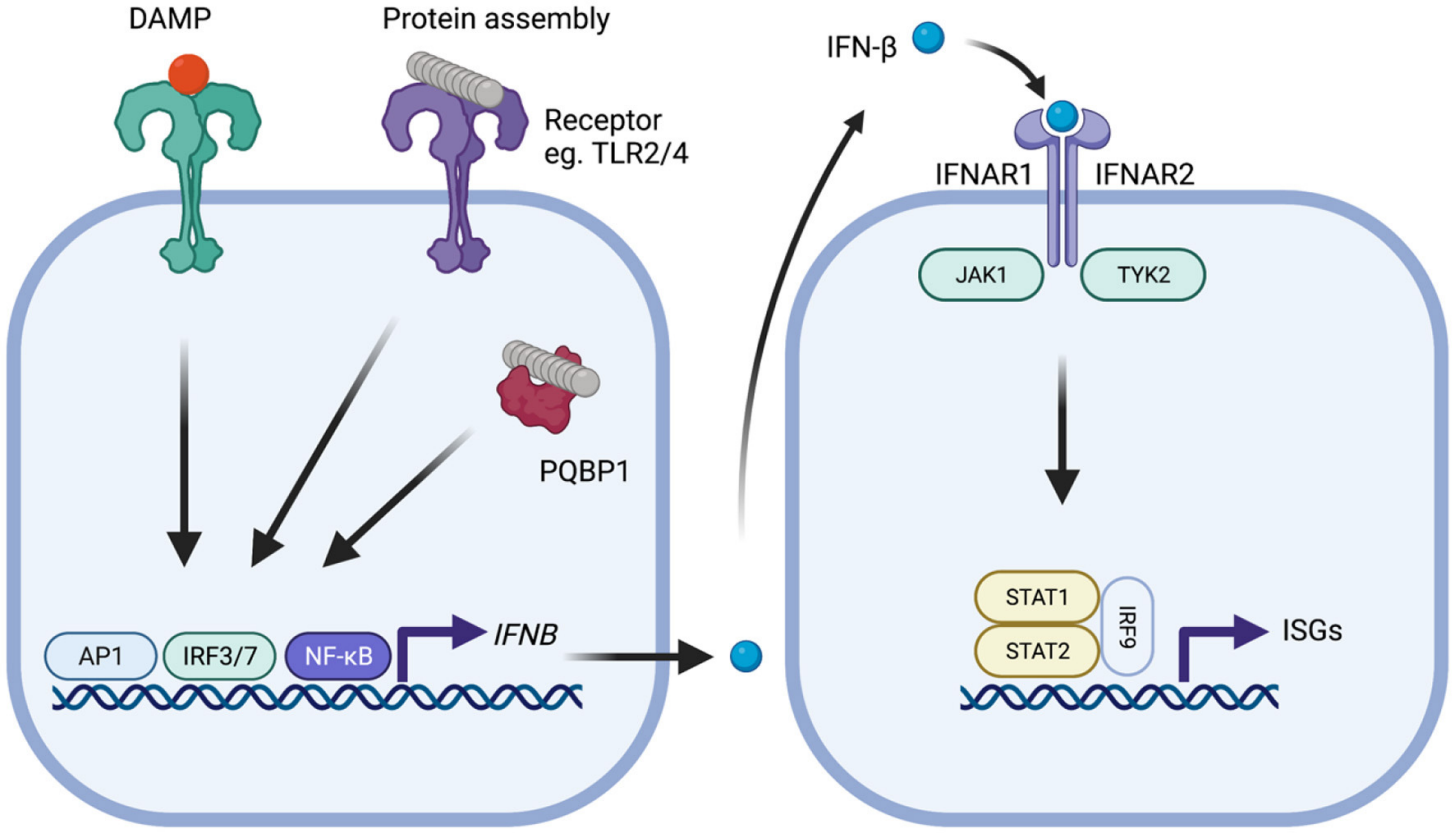
Type-I IFN signaling in the context of neurodegenerative disease. Protein assemblies such as β-amyloid and tau activate innate immune signaling through interactions with cell surface receptors such as TLR2 and TLR4 or intracellular receptors such as PQBP1. Damage associated molecular patterns similarly provoke activation of signaling. Activation of downstream signaling pathways, notably activator protein-1 (AP1), interferon regulatory factor 3 (IRF3), or IRF7 and nuclear factor-kappa B (NF-kB), leads to production of IFN-β which subsequently binds to the type-I IFN receptor complex comprising IFNAR1 and IFNAR2 that is expressed on all nucleated cells. Following type-I IFN binding, the IFNAR receptor complex initiates signaling through the adaptor kinases JAK1 and TYK2 leading to activation of STAT1/STAT2/IRF9 heteromultimers. This complex, referred to as ISGF3, migrates to the nucleus and induces transcription of interferon-stimulated genes (ISGs) that possess interferon-sensitive response element (ISRE). The protein products of these genes help establish an antiviral state and include several innate immune sensors.

Microglia are a critical component of the brain's innate immune response, the first line of defense against foreign pathogens during infection. This response limits the early replication of pathogens while adaptive immune responses are generated (Le Bon and Tough, 2002). Pathogen-associated molecular patterns (PAMPs) or host-derived danger-associated molecular patterns (DAMPs) are recognized by germ-line encoded pattern recognition receptors (PRRs) on the surface of, and inside, host cells (Iwasaki and Medzhitov, 2004; Roh and Sohn, 2018). Microglia are the major site of PRR expression in the brain, though other cell types, particularly astrocytes, also contribute. Engagement of PRRs can result in the transcription of cytokines such as interleukin-1β (IL-1β), interleukin-6 (IL-6), tumor necrosis factor (TNF) and type-I interferons. Each of these have specific roles, including recruitment of professional immune cells and the orchestration of the adaptive immune response. Type-I IFNs comprise IFN-α, of which there are 12 to 13 subtypes, and IFN-β and as well as IFN-ε, IFN-κ, IFN-ω, IFN-δ, IFN-ζ, and IFN-τ. The type-I IFNs have a critical role in inducing an antiviral state in infected and neighboring cells. They exert this function by binding to the type I IFN receptor complex (IFNAR) and initiating signaling through kinases JAK1 and TYK2 which in turn phosphorylate STAT1 and STAT2 transcription factors (Figure 1). This leads to the upregulation of around 2,000 genes in humans, a response that is conserved across mammals (Shaw et al., 2017). As well as their antiviral effects, type-I IFNs have a further role in dampening pro-inflammatory cytokines (Prinz et al., 2008; Goldmann et al., 2016). Each of the major cell types of the CNS retain the ability to both produce and respond to type-I IFN (Supplementary Tables 1–3), though astrocytes and microglia are considered the major sources of production (Scheu et al., 2019).

Inflammation is a key feature of the degenerating brain. In AD, inflammatory markers, including TNF, IL-1β and type-I IFN precede the appearance of symptoms (Taylor et al., 2014; Taipa et al., 2019) and chronically activated, dysfunctional microglia are widespread in the post-mortem brain (Tarkowski, 2003; Navarro et al., 2018). Reactive microglia can be found associated with sites of both tau and Aβ pathologies (Serrano-Pozo et al., 2011). In tauopathies that do not feature Aβ plaques, activated microglia increase proportionately with tau pathology and are found in close proximity to sites of aggregation (Paulus et al., 1993; Gerhard et al., 2006; Malpetti et al., 2020). It is now understood that prolonged immune activation can exacerbate protein aggregation in AD and tauopathies [reviewed by Wyss-Coray and Mucke (2002) and Laurent et al. (2018)]. As major players in the response to infection, and in the degenerating brain, the role of type-I IFNs has come under scrutiny.

## Type-I IFN Signature of Alzheimer's Disease and Tauopathies

Transcripts of IFNα/β and downstream IFN-stimulated genes (ISGs), are significantly higher in brains of AD and clinical dementia patients compared to controls (Taylor et al., 2014; Roy et al., 2020). The expression of IRF7, a transcription factor regulating type-I IFN production, and itself an ISG, is strongly correlated with Braak stage and clinical progression. This elevated IRF7 expression is reflected at the protein level in the prefrontal cortex of AD patients (Taylor et al., 2014; Roy et al., 2020). Larger scale transcriptomic approaches in AD and other tauopathies also show that there is a complex signature of type-I IFN-mediated immune suppression and activation (Rexach et al., 2020). Genome wide association studies (GWAS) implicate genetic variation in innate immune response pathways as important contributors for AD and tau-associated dementias. Polymorphisms in several innate immune genes, including ISGs, are associated with AD risk (Salih et al., 2019). This includes OAS1, a cytosolic RNA sensor responsible for degrading cellular and viral RNAs (Magusali et al., 2021). A type-I IFN signature is therefore a key characteristic in AD and other tauopathies with a potential disease-modifying role.

## Protein Pathologies and Aging Contribute to a Type-I IFN Signature

Protein assemblies are themselves agonists of the type-I IFN response. Aβ_1−42_ assemblies stimulate IFNα/β production in primary neurons, as well as in glial and choroid plexus epithelial cell cultures (Taylor et al., 2014; Mesquita et al., 2015; Minter et al., 2016). Pattern recognition receptors such as toll-like receptor 2 (TLR2) and TLR4, have been identified as receptors for assemblies of Aβ_1−42_ (Liu et al., 2012b; Hughes et al., 2020) and of phosphorylated tau (Meng et al., 2022). This signaling can elicit a downstream IFN response. Tau assemblies can further stimulate type-I IFN via a cytosolic microglial receptor, polyglutamine binding protein 1 (PQBP1) (Jin et al., 2021) and can activate STAT1 (Li et al., 2019) (Table 1). Protein assemblies may therefore be considered as endogenously-derived molecular patterns that provoke innate immune responses similar to PAMPs.

**Table 1:** Summary of studies addressing the production of type-I IFNs in *ex vivo* and *in vitro* models of AD and tauopathy.

**Reference**	**Study design**	**Key observation**
Taylor et al. (2014)	Aβ_1−42_ treated primary neurons from WT or Ifnar1^−/−^ mice	Upregulation of IFN-α/ β. Reduced proinflammatory cytokine production in Ifnar1^−/−^ neurons
Xue et al. (2021)	Oligomeric Aβ-treated organotypic slice cultures	Anti-IFNAR administration reduces Aβ-stimulated microglial phagocytosis of synapses
Roy et al. (2020)	Nucleic acid-amyloid-treated organotypic slice cultures	Secretion of IFN-α/ β. Anti-IFNAR administration reduces nucleic acid-amyloid-stimulated complement C3 expression
Mesquita et al. (2015)	Aβ_1−42_ treated choroid plexus epithelial cells	Upregulation of IFN-α/ β and IFN response genes
Minter et al. (2016)	Aβ_1−42_ treated primary glial cultures	Upregulation of IFN-α/ β. Supernatants from Aβ_1−42_ treated Ifnar1^−/−^ cultures are less neurotoxic and have reduced proinflammatory cytokines
Jin et al. (2021)	Oligomeric/fibrillar tau- treated primary microglia	Upregulation of IFN-α/ β and IFN response genes
Li et al. (2019)	Tau overexpression in HEK293 cells	Increased activation of STAT1. IFN not measured.
Meng et al. (2022)	THP-1 human macrophages treated with hyperphosphorylated tau aggregates	Upregulation of IFN-β and CCL5

In mouse models of Aβ pathology, transcriptomic analysis shows that there is an increased population of IFN-responsive microglia which highly express ISGs (Sala Frigerio et al., 2019; Dorman et al., 2021; Yang et al., 2021). Lineage tracing reveals that these ISG-expressing microglia accumulate progressively in amyloid disease models until a majority of microglia displays evidence of ISG expression (Roy et al., 2022). Similarly, for tau, animal models demonstrate a type-I IFN signature early in the neurodegenerative process (Rexach et al., 2020). Genetic deletion of IFNAR reduces the phagocytic capacity of microglia and dampens the production of pro-inflammatory cytokines in response to Aβ_1−42_. This suggests that type-I IFNs are produced and are important for mediating downstream clearance of aggregates and onward inflammatory events.

Aging itself has been shown to be associated with high levels of type-I IFN in the CNS. Baruch et al. report an age-dependent type-I IFN production at the choroid plexus (Baruch et al., 2014). This has a detrimental effect on cognition which can be reversed by anti-IFNAR antibody administration. Therefore, even in the absence of specific protein pathology, age-related effects contribute to a type-I IFN signature in the brain. These studies suggest that stimuli for the production of type-I IFN likely derive from multiple sources: age-related activation of innate immunity, protein aggregates engaging PRRs and, once disease is established, DAMPs arising from tissue damage related to neurodegeneration. The consequences of chronic type-I IFN production in the CNS on the development of further pathology are not fully elucidated with some apparently contradictory findings. However, accumulating evidence suggests that a IFN response, particularly when chronically produced, is a source of toxicity and potentially exacerbates protein aggregation, thereby setting in motion a destructive feed-forward loop.

## The Role of Type-I IFNS in Disease Pathology

In the periphery, type-I IFNs provide potent protection against infection, yet lead to toxic consequences when over-produced. Dysregulated IFN production leads to severe disease states, exemplified by inherited interferonopathies which have symptoms that mirror viral infection (Crow and Stetson, 2021). In neurodegeneration, type-I IFN is emerging as a central mediator of cascading toxic consequences. Loss of type-I IFN signaling is protective in APP/PS1 mice and 5xFAD mice, alleviating synapse loss and microglial activation (Minter et al., 2016; Roy et al., 2020, 2022) (Table 2). Consistent with this model, administration of IFN-β to WT mice promotes microglial activation, neurotoxicity and synapse loss (Roy et al., 2020). In transgenic mice expressing human tau, cognitive impairment is ameliorated when STAT1 signaling is blocked (Li et al., 2019). Beyond a model of toxicity, further evidence suggests that type-I IFNs can promote further protein aggregation. In mouse models, β-amyloid pathology is ameliorated under conditions of *Ifnar* genetic deletion (Roy et al., 2020, 2022). The effect of IFN on tau aggregation remains unclear, though agonists of the IFN response such as LPS exacerbate pathology (Lee et al., 2010). This raises the prospect that an inappropriate innate immune response to protein aggregates sets in motion a destructive feed-forward loop by inducing further protein aggregation via type-I IFN.

**Table 2:** Summary of studies evaluating type-I IFNs in *in vivo* models of AD and tauopathy.

**References**	**Study design**	**Key observation**	**Type-I IFN: protective/harmful in disease?**
Taylor et al. (2014)	Chimaeric mouse/human APP (Swedish mutation) and mutant human Presenilin overexpression in mice (APP/PS1)	Upregulation of IFN-α in whole brain	
Xue et al. (2021)	Human APP and presenilin with five AD-linked mutations overexpressed in mice (5xFAD)	Upregulation of IFN signaling in microglia	
Roy et al. (2020)	5XFAD, APP/PS1 mice and knock-in humanized APP mice with three AD-linked mutations (APP^NL−G−F^)	Upregulation of IFN response genes in hippocampus	
Mesquita et al. (2015)	Human APP (Swedish and Iberian mutations) overexpression in mice (J20)	Upregulation of IFN response genes in choroid plexus	
Rexach et al. (2020)	Human tau (P301L mutation) overexpression in mice (Tg4510)	Phosphorylated tau pathology correlates with transcription of IFN response genes	
Li et al. (2019)	WT mice overexpressing human tau	Upregulation of phosphorylated STAT1. Blocking STAT1 signaling ameliorates synapse loss and cognitive impairment	Harmful
Minter et al. (2016)	Ifnar1^−/−^ APP/PS1 mice	Ifnar1^−/−^ reduces microgliosis, proinflammatory cytokine secretion and ameliorates cognitive impairment	Harmful
Ejlerskov et al. (2015)	IFN-β^−/−^ and WT mice	Increased neuronal apoptosis and cognitive impairment in IFN-β^−/−^ mice. Lewy bodies containing phosphorylated tau are increased in IFN-β^−/−^ mice	Protective
Chavoshinezhad et al. (2019)	IFN-β administration to APP overexpressing mice (lentivirus)	IFN-β alleviates memory impairments and reduces proinflammatory cytokines (IL-1β, TNFα)	Protective
Mudò et al. (2019)	IFN-β administration to Aβ_1−42_ injected rats	IFN-β alleviates memory impairments and reduces proinflammatory cytokine production induced by Aβ_1−42_	Protective
Roy et al. (2020)	IFN-β administration to WT mice	IFN-β increases synapse loss and microglial activation	Harmful
Roy et al. (2020)	Anti-IFNAR administration to 5xFAD mice	Anti-IFNAR administration alleviates synapse loss and microglial activation	Harmful
Roy et al. (2022)	Ifnar1 conditional knockout (cKO) in 5xFAD mice	Ifnar1^−/−^ cKO in microglia reduces post-synaptic loss; cKO in neurons reduces Aβ plaque accumulation	Harmful
Barnett et al. (2022)	Human APP (Swedish), Mutated tau and presenillin overexpressing mice (3xTg-AD)	pTau181 and Aβ_1−42_ correlate strongly with IFNα in the hippocampus of mice with adolescent intermittent ethanol	

Despite these multiple toxic effects of type-I IFN in the CNS, the view that its production is universally detrimental is not supported by other findings, particularly in acute settings. APP overexpression by lentiviral transduction or Aβ_1−42_ peptide delivery to the mouse brain induces pro-inflammatory cytokine production. This can be reduced by administration of IFN-β, consistent with the anti-inflammatory effects of type-I IFN (Chavoshinezhad et al., 2019; Mudò et al., 2019). Furthermore, genetic knockout of IFN-β is associated with reduced autophagic clearance and accumulation of ubiquitinated alpha-synuclein aggregates in mice (Ejlerskov et al., 2015). One possibility is that IFNs, while protective in acute settings, lead to damage when chronically over-produced, consistent with the “double-edged sword” hypothesis of innate immune activation in neurodegeneration (Wyss-Coray and Mucke, 2002). This would broadly align with our understanding of IFN in peripheral infection: that IFNs are highly protective when appropriately expressed yet can unleash severe damage when dysregulated or chronically over-produced.

## Type-I IFNS in Models of Prion and Parkinson's Disease

Interesting parallels for tauopathies can be sought by examining other protein misfolding diseases of the CNS. Parkinson's disease (PD) is characterized by the aggregation of the cytosolic protein alpha-synuclein. Prion diseases such as Creutzfeld-Jakob disease in humans and scrapie in other animals are driven by the conversion of a membrane-anchored protein, PrP, to a misfolded variant. Genetic depletion of signaling components such as IRF3, Ifnar, TLR4 and TLR2 render mice more susceptible to the scrapie variant of PrP, PrP^Sc^. This suggests a protective role for type-I IFNs in the recognition and control of prion assemblies (Ishibashi et al., 2012, 2019; Carroll et al., 2018). However, even here, type-I IFN production comes at a cost, as Nazmi et al. show that neuronal death is accelerated by Ifnar-dependent signaling (Nazmi et al., 2019).

In PD, type-I IFN and ISG transcripts are upregulated, similar to observations in AD and other tauopathies (Main et al., 2016). The effects of type-I IFN appear to be model-dependent. In one model of PD, neuronal loss is induced by 1-methyl-4-phenyl-1, 2, 3, 6-tetrahydropyridine (MPTP) injection. Blockade of IFN signaling using anti-Ifnar antibodies supressed dopaminergic neuronal death, suggesting that type-I IFN signaling is neurotoxic (Main et al., 2016). In contrast, genetic deletion of IFN-β caused the formation of α-syn-positive Lewy body structures and reduced autophagic clearance (Ejlerskov et al., 2015). As in the tauopathies, these findings again point to production of type-I IFN having an important role in protection against protein aggregation, but with over-production contributing to toxicity. Any therapeutic intervention by manipulation of the type-I IFN pathway in proteopathies must therefore seek to target the over-production of IFN whilst ensuring that its essential functions in the control of proteinopathy are not unduly compromised.

## Discussion

Type-I IFN plays a critical role in the brain during pathogen infection. Like classical pathogens, aggregated proteins including Aβ and tau can provoke an immune reaction that includes the production of type-I IFN. *In vivo*, the literature lacks clarity on whether IFNs are harmful or beneficial, likely owing to the timescales over which protection is measured. Protective effects of type-I IFN have been observed for Aβ, prion and α-syn especially in short-term challenge experiments, suggesting that common protective mechanisms may be at play. However, type-I IFNs promote downstream toxic consequences which may be amplified in a positive-feedback manner in response to ongoing tissue damage and further protein aggregation. Our understanding of these effects is in its infancy and remains largely without mechanistic detail. Further, for tau pathology, there remains little insight to the effect of IFN signaling due to the lack of studies using genetic knockout or experimental IFN-blockade. Future research should seek to dissect the IFN response at the level of specific ISGs to identify those that aid in limiting protein aggregation vs. those that promote toxic downstream consequences. An understanding at this level may allow selective pharmacological intervention to prevent the chronic toxic consequences of IFN signaling in neurodegeneration.

## Author Contributions

SS and WM wrote and edited the manuscript. SS collated information used in the tables and supplementary tables. Both authors contributed to the article and approved the submitted version.

## Funding

SS was funded by Alzheimer's Society Ph.D. Studentship, Grant Reference 488. WM was a Lister Institute Fellow and supported by a Sir Henry Dale Fellowship jointly funded by the Wellcome Trust and the Royal Society (206248/Z/17/Z). This work was supported by the UK Dementia Research Institute, which receives its funding from DRI Ltd., funded by the UK Medical Research Council, Alzheimer's Society, and Alzheimer's Research UK.

## Conflict of Interest

The authors declare that the research was conducted in the absence of any commercial or financial relationships that could be construed as a potential conflict of interest.

## Publisher's Note

All claims expressed in this article are solely those of the authors and do not necessarily represent those of their affiliated organizations, or those of the publisher, the editors and the reviewers. Any product that may be evaluated in this article, or claim that may be made by its manufacturer, is not guaranteed or endorsed by the publisher.
