# Supplementary material for: Type-I Interferons in Alzheimer's Disease and Other Tauopathies
Source: Front Cell Neurosci. 2022 Jul 15;16:949340. doi: 10.3389/fncel.2022.949340 (PMC9334774; doi:10.3389/fncel.2022.949340)
Supplement: Supplementary file 1 [file Table_1.DOCX]

**Supplementary Table 1:** Summary of studies evaluating Type-I IFN responses and production in neurons

| Marker | Production of  type-I IFN | | |  | Response to  type-I IFN | | *Neuron type* | Reference |
| --- | --- | --- | --- | --- | --- | --- | --- | --- |
|  | *Species* | *Agonist* | *Validation* |  | *Species* | *Readout* |  |  |
| NeuN+ | Murine | TMEV/ LACV | Protein |  | Murine | ISG protein |  | Delhaye, S. et al. 2006 ^1^ |
| MAP2+ |  |  |  |  | Murine | ISG transcripts/MHV replication | Cortical/Granule cell | Cho H. et al. 2013 ^2^ |
| NeuF-H | Human | RABV/ Poly(I:C) | mRNA/ Protein |  | Human | ISG transcripts | NT2-N cells | Prehaud, C., Megret, F., Lafage, M. & Lafon, M. 2005^3^ |
| MAP2+ |  |  |  |  | Rat | ISG transcripts | Hippocampal/cortical | Kreit, M. et al. 2014^4^ |
| NeuN+ |  |  |  |  | Murine | ISG protein/HSV1 replication |  | Reinert, L. S. et al. 2016^5^ |
| NeuN+ | Murine |  | Protein |  | Murine | IFNb-/- mice, neuronal replication |  | Ejlerskov, P. et al. 2015^6^ |
| Nestin+ |  |  |  |  | Murine | Proliferation (BrdU+) | Hippocampal neural stem cells | Zheng, L. S. et al. 2014 ^7^ |
| Nissl+ |  |  |  |  | Murine | ISG transcripts | Hippocampal/cerebellar neurons | Wang, J., Campbell, I. L. & Zhang, H. 2008^8^ |
| NeuN+ | Murine | Aβ_1-42_ peptide | mRNA |  | Human, Murine | IFNAR-/- neurons, neuronal viability | M17 cells (Hu) | Taylor, J. M. et al. 2014^9^ |
|  |  |  |  |  | Rat | Electrophysiology | Hippocampal CA3 pyramidal neurons | Müller, M., Fontana, A., Zbinden, G. & Gähwiler, B. H. 1993^10^ |
|  |  |  |  |  | Rat | Electrophysiology | Hippocampal CA1 pyramidal neurons | Mendoza-Fernández, V., Andrew, R. D. & Barajas-López, C. 2000^11^ |
|  |  |  |  |  | Murine | Electrophysiology | Striatal medium-sized projecting spiny neurons (MSNs) | Di Filippo, M. *et al.* 2016^12^, Di Filippo, M. *et al.* 2014^13^ |
| HuC+/ HuD+ | Human | TBEV | mRNA |  | Human | ISG transcripts | Differentiated neural progenitor cells | Fares, M. *et al.* 2020^14^ |
|  | Murine | VSV | Protein |  | Murine | ISG transcripts/HSV1 replication | Primary neurons | Yordy, B., Iijima, N., Huttner, A., Leib, D. & Iwasaki, A. 2020^15^ |
| MAP2+ |  |  |  |  | Murine | Dendritic aborisation | Primary cortical neurons | Sas, A. R., Bimonte-Nelson, H., Smothers, C. T., Woodward, J. & Tyor, W. R. 2009^16^ |
| Nestin2/ TUJ11+ | Human | HSV1/ Poly(I:C) | mRNA |  | Human | ISG transcripts | iPSC-derived NSCs | Lafaille, F. G. *et al.* 2012^17^ |
| NeuN+ | Human | IFN-λ | mRNA |  | Human | ISG transcripts | Primary human neurons | Li, J. *et al.* 2011^18^ |
| NeuN+ |  |  |  |  | Murine | ISG transcripts/HSV1 replication | Trigeminal ganglia (TG) neurons | Rosato, P. C. & Leib, D. A. 2015^19^ |
| MAP2+ |  | MHV/ SeV | mRNA |  |  |  |  | Roth-Cross, J. K., Bender, S. J. & Weiss, S. R. 2008^20^ |
|  |  |  |  |  | Murine | pNR2B expression, electrophysiology | Hippocampal CA1 Pyramidal Neurons | Costello, D. A. & Lynch, M. A. 2013^21^ |
|  |  |  |  |  | Rat, Murine | IFNAR-/- neurons, cell viability/ISG transcripts | Cerebrocortical cultures | Thaney, V. E. *et al.* 2017^22^ |
|  |  |  |  |  | Rat | Electrophysiology | Pyramidal Neurons | Hadjilambreva, G., Mix, E., Rolfs, A., Müller, J. & Strauss, U. 2005^23^ |
| Nestin+ |  |  |  |  |  | ISG transcripts/IFNAR expression | Neural progenitor cells | Hirsch, M. *et al.* 2009^24^ |
| tubulin+, TH+ |  |  |  |  | Human | ISG transcripts | hESC derived neurons | Massey, A. R. *et al.* 2020^25^ |
| Dcx+, NeuN+ |  |  |  |  | Primate (Marmoset) | Neuronal proliferation | Hippocampal dentate gyrus | Kaneko, N., Nakamura, S. & Sawamoto, K. 2020^26^ |
| MAP2+ |  |  |  |  | Murine | ISG transcripts/pSTAT1 expression, IFNAR-/- neurons | Hippocampal neurons | Cavanaugh, S. E., Holmgren, A. M. & Rall, G. F. ^27^ |
| β-tubulin III |  |  |  |  | Murine | IFITM3 protein expression/γ-secretase activity | Primary neurons | Hur et al. 2020 ^28^ |
| Nestin+ |  |  |  |  | Murine | Selective Ifnar depletion (Nestin-cre x Ifnar1fl/fl, ISG (IFITM3/STAT1) expression, synapse loss | All neuroectodermal-derived cells | Roy et al. 2022^29^ |

ISG: Interferon Stimulated Gene; NT2-N: Human neuroblastoma cell line; iPSC: Induced Pluripotent Stem Cell; hESC: human embryonic stem cell

1. Delhaye, S. *et al.* Neurons produce type I interferon during viral encephalitis. *Proc. Natl. Acad. Sci. U. S. A.* (2006) doi:10.1073/pnas.0602460103.

2. Cho, H. *et al.* Differential innate immune response programs in neuronal subtypes determine susceptibility to infection in the brain by positive-stranded RNA viruses. *Nat. Med.* (2013) doi:10.1038/nm.3108.

3. Prehaud, C., Megret, F., Lafage, M. & Lafon, M. Virus Infection Switches TLR-3-Positive Human Neurons To Become Strong Producers of Beta Interferon. *J. Virol.* **79**, 12893–12904 (2005).

4. Kreit, M. *et al.* Inefficient Type I Interferon-Mediated Antiviral Protection of Primary Mouse Neurons Is Associated with the Lack of Apolipoprotein L9 Expression. *J. Virol.* **88**, 3874–3884 (2014).

5. Reinert, L. S. *et al.* Sensing of HSV-1 by the cGAS-STING pathway in microglia orchestrates antiviral defence in the CNS. *Nat. Commun.* **7**, 1–12 (2016).

6. Ejlerskov, P. *et al.* Lack of Neuronal IFN-β-IFNAR Causes Lewy Body- and Parkinson’s Disease-like Dementia. *Cell* **163**, 324–339 (2015).

7. Zheng, L. S. *et al.* Mechanisms for interferon-α-induced depression and neural stem cell dysfunction. *Stem Cell Reports* **3**, 73–84 (2014).

8. Wang, J., Campbell, I. L. & Zhang, H. Systemic interferon-α regulates interferon-stimulated genes in the central nervous system. *Mol. Psychiatry* **13**, 293–301 (2008).

9. Taylor, J. M. *et al.* Type-1 interferon signaling mediates neuro-inflammatory events in models of Alzheimer’s disease. *Neurobiol. Aging* **35**, 1012–1023 (2014).

10. Müller, M., Fontana, A., Zbinden, G. & Gähwiler, B. H. Effects of interferons and hydrogen peroxide on CA3 pyramidal cells in rat hippocampal slice cultures. *Brain Res.* **619**, 157–162 (1993).

11. Mendoza-Fernández, V., Andrew, R. D. & Barajas-López, C. Interferon-α inhibits long-term potentiation and unmasks a long-term depression in the rat hippocampus. *Brain Res.* **885**, 14–24 (2000).

12. Di Filippo, M. *et al.* Interferon-β1a modulates glutamate neurotransmission in the CNS through CaMKII and GluN2A-containing NMDA receptors. *Neuropharmacology* **100**, 98–105 (2016).

13. Di Filippo, M. *et al.* Interferon-β1a protects neurons against mitochondrial toxicity via modulation of STAT1 signaling: Electrophysiological evidence. *Neurobiol. Dis.* **62**, 387–393 (2014).

14. Fares, M. *et al.* Pathological modeling of TBEV infection reveals differential innate immune responses in human neurons and astrocytes that correlate with their susceptibility to infection. *J. Neuroinflammation* **17**, 76 (2020).

15. Yordy, B., Iijima, N., Huttner, A., Leib, D. & Iwasaki, A. A neuron-specific role for autophagy in antiviral defense against herpes simplex virus. *Cell Host Microbe* **12**, 334–345 (2012).

16. Sas, A. R., Bimonte-Nelson, H., Smothers, C. T., Woodward, J. & Tyor, W. R. Interferon-α causes neuronal dysfunction in encephalitis. *J. Neurosci.* **29**, 3948–3955 (2009).

17. Lafaille, F. G. *et al.* Impaired intrinsic immunity to HSV-1 in human iPSC-derived TLR3-deficient CNS cells. *Nature* **491**, 769–773 (2012).

18. Li, J. *et al.* Interferon lambda inhibits herpes simplex virus type I infection of human astrocytes and neurons. *Glia* **59**, 58–67 (2011).

19. Rosato, P. C. & Leib, D. A. Neuronal Interferon Signaling Is Required for Protection against Herpes Simplex Virus Replication and Pathogenesis. *PLoS Pathog.* **11**, (2015).

20. Roth-Cross, J. K., Bender, S. J. & Weiss, S. R. Murine Coronavirus Mouse Hepatitis Virus Is Recognized by MDA5 and Induces Type I Interferon in Brain Macrophages/Microglia. *J. Virol.* **82**, 9829–9838 (2008).

21. Costello, D. A. & Lynch, M. A. Toll-like receptor 3 activation modulates hippocampal network excitability, via glial production of interferon-beta. *Hippocampus* **23**, 696–707 (2013).

22. Thaney, V. E. *et al.* IFNβ Protects Neurons from Damage in a Murine Model of HIV-1 Associated Brain Injury. *Sci. Rep.* **7**, 46514 (2017).

23. Hadjilambreva, G., Mix, E., Rolfs, A., Müller, J. & Strauss, U. Neuromodulation by a cytokine: Interferon-β differentially augments neocortical neuronal activity and excitability. *J. Neurophysiol.* **93**, 843–852 (2005).

24. Hirsch, M. *et al.* The effect of interferon-β on mouse neural progenitor cell survival and differentiation. *Biochem. Biophys. Res. Commun.* **388**, 181–186 (2009).

25. Massey, A. R. *et al.* Alpha-synuclein expression supports interferon stimulated gene expression in neurons. *bioRxiv* 2020.04.25.061762 (2020) doi:10.1101/2020.04.25.061762.

26. Kaneko, N., Nakamura, S. & Sawamoto, K. Effects of interferon-alpha on hippocampal neurogenesis and behavior in common marmosets. *Mol. Brain* **13**, 98 (2020).

27. Cavanaugh, S. E., Holmgren, A. M. & Rall, G. F. Homeostatic interferon expression in neurons is sufficient for early control of viral infection. *J. Neuroimmunol.* **279**, 11–19 (2015).

28. Hur, J. Y. *et al.* The innate immunity protein IFITM3 modulates γ-secretase in Alzheimer’s disease. *Nature* (2020) doi:10.1038/s41586-020-2681-2.

29. Roy, E. R. *et al.* Concerted type I interferon signaling in microglia and neural cells promotes memory impairment associated with amyloid β plaques. *Immunity* 1–16 (2022) doi:10.1016/j.immuni.2022.03.018.
