# Supplementary material for: Type-I Interferons in Alzheimer's Disease and Other Tauopathies
Source: Front Cell Neurosci. 2022 Jul 15;16:949340. doi: 10.3389/fncel.2022.949340 (PMC9334774; doi:10.3389/fncel.2022.949340)
Supplement: Supplementary file 2 [file Table_2.DOCX]

**Supplementary Table 2:** Summary of studies evaluating Type-I IFN responses and production in microglia

| Marker | Production of  type-I IFN | | |  | Response to  type-I IFN | | Reference |
| --- | --- | --- | --- | --- | --- | --- | --- |
|  | *Species* | *Agonist* | *Validation* |  | *Species* | *Readout* |  |
| Iba1+, CD11b+/CD45int or CD45hi | Murine | Poly(I:C) | mRNA (reporter) |  | Murine | Phagocytosis capacity/ associated gene transcripts | Kocur, M. *et al.* 2015 ^1^ |
| Iba1+ | Human | Age | Protein |  | Murine | ISG transcripts/Iba1+ expression | Baruch, K. *et al.* 2014^2^ |
| CD45+ | Murine | HSV1 | mRNA/ Protein |  |  |  | Reinert, L. S. *et al.* 2016^3^ |
| CD11b+/CD45+ | Murine | Poly(I:C) | mRNA (reporter) |  |  | ISG transcripts | Khorooshi, R. *et al.* 2015^4^ |
| Iba1+ | Murine | IFN-α | Protein |  | Murine | Cytokine transcripts/Iba1+ expression | Zheng, L. S., Kaneko, N. & Sawamoto, K. 2015^5^ |
| Iba1+ |  |  |  |  | Rat | Iba1+ expressing cells | Bi, Q., Shi, L., Yang, P., Wang, J. & Qin, L. 2016^6^ |
| Iba1+ |  |  |  |  | Murine | Usp18 mRNA/protein expression, ISG transcripts | Goldmann, T. et al. 2015^7^ |
| Morphology | Murine | EAE model | Protein |  |  |  | Teige, I. *et al.* 2003^8^ |
| CD45+ (LCA) |  |  |  |  | Human | IFNAR, ISG protein expression | Yamada, T., Horisberger, M. A., Kawaguchi, N., Moroo, I. & Toyoda, T. (1994), Yamada, T. & Yamanaka, I. (1995)^9,10^ |
| HAM56+ or CD68+ |  |  |  |  | Human | MIP1α/β, RANTES, MCP mRNA and protein expression | McManus, C. M. *et al.* (2000)^11^ |
| GS-1 lectin+ |  |  |  |  | Rat | IL-1 protein secretion, chemotaxis | Colton, C. A., Yao, J., Keri, J. E. & Gilbert, D. (1992)^12^ |
| CD68+ |  |  |  |  | Human | NFkB activation, STAT1/ERK phosphorylation, MIP1α/β, RANTES protein secretion | Kim, M.-O. *et al.* (2002)^13^ |
| FcR+ |  |  |  |  | Murine | Cytokine secretion | Jin, S. *et al.* (2007)^14^ |
| CD11b int/ CD45int | Murine | MHV/ SeV | mRNA |  |  |  | Roth-Cross, J. K., Bender, S. J. & Weiss, S. R. (2008)^15^ |
| CD45+ | Human | AD (?) | Protein |  |  |  | Akiyama, H., Ikeda, K., Katoh, M., McGeer, E. G. & McGeer, P. L (1994)^16^ |
| FcR+ |  |  |  |  | Murine | Cytokine secretion, antigen presenting function, MHC class II expression | Kawanokuchi, J., Mizuno, T., Kato, H., Mitsuma, N. & Suzumura, A. (2004)^17^ |
|  |  |  |  |  | Murine | Respiratory burst, MHC class II expression | Hall, G. L., Wing, M. G., Compston, D. A. S. & Scolding, N. J. (1997)^18^ |
| CD11b+ | Murine | Ganciclovir | mRNA/ protein |  |  |  | Mathur, V. *et al.* (2017)^19^ |
| CD11b+ | Murine | NSV, Poly(I:C) | Protein |  | Murine | CXCL13 expression | Esen, N., Rainey-Barger, E. K., Huber, A. K., Blakely, P. K. & Irani, D. N. (2014)^20^ |
|  | Murine | LPS | mRNA/ protein |  |  |  | Kozela, E. *et al.* (2010)^21^ |
| CD68+ | Human | Poly(I:C) | Protein |  |  |  | Tarassishin, L., Suh, H.-S. S. & Lee, S. C. (2011)^22^ |
| F4/80+ | Murine | LACV | mRNA (reporter) |  |  |  | Kallfass, C. *et al.* (2012)^23^ |
|  | Human | JEV | mRNA (reporter) |  |  |  | Manocha, G. D. *et al.* (2014)^24^ |
| Iba1+/ CD11b+ |  |  |  |  | Murine | IFNAR-/- mice, MeV infection | Welsch, J. C. *et al.* (2019)^25^ |
|  | Murine | Poly(I:C) | mRNA/ protein |  |  |  | Costello, D. A. & Lynch, M. A. (2013)^26^ |
| Iba1+ |  |  |  |  | Murine | Iba1+ protein expression | Thaney, V. E. *et al.* (2017)^27^ |
|  | Murine, Human | HSV1 | mRNA |  |  |  | Bodda, C. *et al.* (2020)^28^ |
| CD11b+ | Human | S. aureus | Protein |  |  |  | Johnson, M. B. *et al.* (2020)^29^ |
| CD11b+/CD45+ | Murine | Prion | mRNA |  | Murine | IFNAR-/- mice, ISG transcripts | Nazmi, A. *et al.* (2019)^30^ |
| CD11bintCD45int or CD11bintCD45intCX3CR1+ | Murine | Poly(I:C) | mRNA |  | Murine | IFNAR-/- mice, microglial proliferation | Ben-Yehuda, H. *et al.* (2020)^31^ |
| CD11b+/CD45int |  |  |  |  | Murine | ISG transcripts | Todd et al. 2021^32^ |
| n/a | Murine | Tau | mRNA |  | Murine | ISG transcripts | Jin et al. 2021^33^ |
| CX3CR1+/ CD11b+ | Murine | Aβ_1-42_ | mRNA/ Protein |  | Human/Murine | Phagocytosis, Ifnar-/- microglia, cytokine expression | Moore et al. 2020^34^ |
| CD11b+/NeuN- /GFAP- |  |  |  |  | Murine | ISG transcripts, anti-IFNAR antibody | Xue et al. 2021^35^ |
| Iba1+/ CX3CR1+ | Murine | Human mutant APP | mRNA/Protein |  | Murine | ISG transcripts, anti-IFNAR antibody Selective Ifnar depletion in microglia: Cx3cr1-CreERT x Ifnar1^fl/fl^ | Roy et al. 2022^36^ |

1. Kocur, M. *et al.* IFNβ secreted by microglia mediates clearance of myelin debris in CNS autoimmunity. *Acta Neuropathol. Commun.* **3**, 20 (2015).

2. Baruch, K. *et al.* Aging-induced type I interferon response at the choroid plexus negatively affects brain function. *Science (80-. ).* (2014) doi:10.1126/science.1252945.

3. Reinert, L. S. *et al.* Sensing of HSV-1 by the cGAS-STING pathway in microglia orchestrates antiviral defence in the CNS. *Nat. Commun.* **7**, 1–12 (2016).

4. Khorooshi, R. *et al.* Induction of endogenous Type I interferon within the central nervous system plays a protective role in experimental autoimmune encephalomyelitis. *Acta Neuropathol.* **130**, 107–118 (2015).

5. Zheng, L. S., Kaneko, N. & Sawamoto, K. Minocycline treatment ameliorates interferon-alpha-induced neurogenic defects and depression-like behaviors in mice. *Front. Cell. Neurosci.* **9**, (2015).

6. Bi, Q., Shi, L., Yang, P., Wang, J. & Qin, L. Minocycline attenuates interferon-α-induced impairments in rat fear extinction. *J. Neuroinflammation* **13**, 172 (2016).

7. Goldmann, T. *et al.* USP18 lack in microglia causes destructive interferonopathy of the mouse brain. *EMBO J.* **34**, (2015).

8. Teige, I. *et al.* IFN-beta gene deletion leads to augmented and chronic demyelinating experimental autoimmune encephalomyelitis. *J. Immunol.* **170**, 4776–4784 (2003).

9. Yamada, T., Horisberger, M. A., Kawaguchi, N., Moroo, I. & Toyoda, T. Immunohistochemistry using antibodies to α-interferon and its induced protein, MxA, in Alzheimer’s and Parkinson’s disease brain tissues. *Neurosci. Lett.* **181**, 61–64 (1994).

10. Yamada, T. & Yamanaka, I. Microglial localization of α-interferon receptor in human brain tissues. *Neurosci. Lett.* **189**, 73–76 (1995).

11. McManus, C. M. *et al.* Differential induction of chemokines in human microglia by type I and II interferons. *Glia* **29**, 273–280 (2000).

12. Colton, C. A., Yao, J., Keri, J. E. & Gilbert, D. Regulation of microglial function by interferons. *J. Neuroimmunol.* **40**, 89–98 (1992).

13. Kim, M.-O. *et al.* Interferon-β activates multiple signaling cascades in primary human microglia. *J. Neurochem.* **81**, 1361–1371 (2002).

14. Jin, S. *et al.* Interferon-beta is neuroprotective against the toxicity induced by activated microglia. *Brain Res.* **1179**, 140–146 (2007).

15. Roth-Cross, J. K., Bender, S. J. & Weiss, S. R. Murine Coronavirus Mouse Hepatitis Virus Is Recognized by MDA5 and Induces Type I Interferon in Brain Macrophages/Microglia. *J. Virol.* **82**, 9829–9838 (2008).

16. Akiyama, H., Ikeda, K., Katoh, M., McGeer, E. G. & McGeer, P. L. Expression of MRP14, 27E10, interferon-α and leukocyte common antigen by reactive microglia in postmortem human brain tissue. *J. Neuroimmunol.* **50**, 195–201 (1994).

17. Kawanokuchi, J., Mizuno, T., Kato, H., Mitsuma, N. & Suzumura, A. Effects of interferon-β on microglial functions as inflammatory and antigen presenting cells in the central nervous system. *Neuropharmacology* **46**, 734–742 (2004).

18. Hall, G. L., Wing, M. G., Compston, D. A. S. & Scolding, N. J. β-interferon regulates the immunomodulatory activity of neonatal rodent microglia. *J. Neuroimmunol.* **72**, 11–19 (1997).

19. Mathur, V. *et al.* Activation of the STING-Dependent Type I Interferon Response Reduces Microglial Reactivity and Neuroinflammation. *Neuron* **96**, 1290-1302.e6 (2017).

20. Esen, N., Rainey-Barger, E. K., Huber, A. K., Blakely, P. K. & Irani, D. N. Type-I interferons suppress microglial production of the lymphoid chemokine, CXCL13. *Glia* **62**, 1452–1462 (2014).

21. Kozela, E. *et al.* Cannabinoids Δ9-tetrahydrocannabinol and cannabidiol differentially inhibit the lipopolysaccharide-activated NF-κB and interferon-β/STAT proinflammatory pathways in BV-2 microglial cells. *J. Biol. Chem.* **285**, 1616–1626 (2010).

22. Tarassishin, L., Suh, H.-S. S. & Lee, S. C. Interferon regulatory factor 3 plays an anti-inflammatory role in microglia by activating the PI3K/Akt pathway. *J. Neuroinflammation* **8**, 187 (2011).

23. Kallfass, C. *et al.* Visualizing Production of Beta Interferon by Astrocytes and Microglia in Brain of La Crosse Virus-Infected Mice. *J. Virol.* **86**, 11223–11230 (2012).

24. Manocha, G. D. *et al.* Regulatory role of TRIM21 in the type-I interferon pathway in Japanese encephalitis virus-infected human microglial cells. *J. Neuroinflammation* **11**, 24 (2014).

25. Welsch, J. C. *et al.* Type I Interferon Receptor Signaling Drives Selective Permissiveness of Astrocytes and Microglia to Measles Virus during Brain Infection. *J. Virol.* **93**, (2019).

26. Costello, D. A. & Lynch, M. A. Toll-like receptor 3 activation modulates hippocampal network excitability, via glial production of interferon-beta. *Hippocampus* **23**, 696–707 (2013).

27. Thaney, V. E. *et al.* IFNβ Protects Neurons from Damage in a Murine Model of HIV-1 Associated Brain Injury. *Sci. Rep.* **7**, 46514 (2017).

28. Bodda, C. *et al.* HSV1 VP1-2 deubiquitinates STING to block type I interferon expression and promote brain infection. *J. Exp. Med.* **217**, (2020).

29. Johnson, M. B. *et al.* Retinoic acid inducible gene-I mediated detection of bacterial nucleic acids in human microglial cells. *J. Neuroinflammation* **17**, 1–14 (2020).

30. Nazmi, A. *et al.* Chronic neurodegeneration induces type I interferon synthesis via STING, shaping microglial phenotype and accelerating disease progression. *Glia* (2019) doi:10.1002/glia.23592.

31. Ben-Yehuda, H. *et al.* Maternal Type-I interferon signaling adversely affects the microglia and the behavior of the offspring accompanied by increased sensitivity to stress. *Mol. Psychiatry* **25**, 1050–1067 (2020).

32. Todd, B. P. *et al.* Traumatic brain injury results in unique microglial and astrocyte transcriptomes enriched for type I interferon response. *J. Neuroinflammation 2021 181* **18**, 1–15 (2021).

33. Jin, M. *et al.* Tau activates microglia via the PQBP1-cGAS-STING pathway to promote brain inflammation. *Nat. Commun.* **12**, 6565 (2021).

34. Moore, Z., Mobilio, F., Walker, F. R., Taylor, J. M. & Crack, P. J. Abrogation of type-I interferon signalling alters the microglial response to Abeta1-42. *Sci. Rep.* **10**, 3153 (2020).

35. Xue, F., Tian, J., Yu, C., Du, H. & Guo, L. Type I interferon response-related microglial Mef2c deregulation at the onset of Alzheimer’s pathology in 5×FAD mice. *Neurobiol. Dis.* **152**, 105272 (2021).

36. Roy, E. R. *et al.* Concerted type I interferon signaling in microglia and neural cells promotes memory impairment associated with amyloid β plaques. *Immunity* 1–16 (2022) doi:10.1016/j.immuni.2022.03.018.
