# Supplementary material for: Type-I Interferons in Alzheimer's Disease and Other Tauopathies
Source: Front Cell Neurosci. 2022 Jul 15;16:949340. doi: 10.3389/fncel.2022.949340 (PMC9334774; doi:10.3389/fncel.2022.949340)
Supplement: Supplementary file 3 [file Table_3.DOCX]

**Supplementary Table 3:** Summary of studies evaluating Type-I IFN responses and production in astrocytes

| Marker | Production of  type-I IFN | | |  | Response to  type-I IFN | | Reference |
| --- | --- | --- | --- | --- | --- | --- | --- |
|  | *Species* | *Agonist* | *Validation* |  | *Species* | *Readout* |  |
| n/a | Murine | TMEV/LACV | Protein |  | Murine | ISG protein | Delhaye, S. et al. 2006 ^1^ |
| GFAP+ | Rat/ Murine |  |  |  | Murine | ISG (Mx1) transcripts/protein/VSV-GFP infection | Lin et al. 2013^2^ |
| GFAP+ | Murine/ Human | Age | Protein |  | Murine | ISG transcripts/GFAP+ expression | Baruch, K. *et al.* 2014^3^ |
| GFAP+ | Murine | HSV1 | mRNA/ Protein |  | Murine | ISG protein/HSV1 replication | Reinert, L. S. et al. 2016^4^ |
| GFAP+ | Murine | Poly(I:C) | mRNA (reporter) |  |  | ISG transcripts | Khorooshi, R. *et al.* 2015^5^ |
| GFAP+ |  |  |  |  | Rat | GFAP+ expressing cells | Bi, Q., Shi, L., Yang, P., Wang, J. & Qin, L. 2016^6^ |
| GFAP+ |  |  |  |  | Murine | ISG transcripts | Wang, J., Campbell, I. L. & Zhang, H. 2008^7^ |
| GFAP+ |  |  |  |  | Murine | ISG transcripts/Ifnar-/- astrocytes | Rothhammer et al. 2016^8^ |
| GFAP+ | Human | TBEV | mRNA |  | Human | ISG transcripts | Fares, M. *et al.* 2020^9^ |
| GFAP+ | Human | HSV1/ Poly(I:C) | mRNA |  | Human | ISG transcripts | Lafaille, F. G. *et al.* 2012^10^ |
| n/a | Human | IFN-λ | mRNA |  | Human | ISG transcripts | Li, J. *et al.* 2011^11^ |
| GFAP+ | Murine | SeV | mRNA |  |  |  | Roth-Cross, J. K., Bender, S. J. & Weiss, S. R. (2008)^12^ |
| n/a | Human | Poly(I:C) | Protein |  |  |  | Tarassishin, L., Suh, H.-S. S. & Lee, S. C. (2011)^13^ |
| GFAP+ | Murine | LACV | mRNA (reporter) |  |  |  | Kallfass, C. *et al.* (2012)^14^ |
| GFAP+ |  |  |  |  | Murine | Ifnar/- mice, MeV infection | Welsch, J. C. *et al.* (2019)^15^ |
| GFAP+ |  |  |  |  | Murine | Cognitive deficits, conditional Ifnar-/- and Ifnb-/- mice | Hosseini et al. 2020^16^ |
| n/a | Murine | Poly(I:C) | mRNA/ protein |  |  |  | Costello, D. A. & Lynch, M. A. (2013)^17^ |
| GFAP+ |  |  |  |  | Rat, Murine | CCL4 secretion | Thaney, V. E. *et al.* 2017^18^ |
| GFAP+ | Human | S. aureus | Protein |  |  |  | Johnson, M. B. *et al.* (2020)^19^ |
| GFAP+ |  |  |  |  | Rat | Apoptosis measurements, NfkB/P13K signalling | Barca et al. 2010^20^ |
| GFAP+ |  |  |  |  | Murine | IFNAR-/- mice, ISG/C3 transcripts | Nazmi, A. *et al.* (2019)^21^ |
| n/a |  |  |  |  | Murine, Human (astrocytoma) | IL-6 expression | Okada et al. 2005^22^ |
| GFAP+ | Murine | TBEV | mRNA |  | Murine | ISG transcripts, Ifnar-/- astrocytes | Lindqvist et al. 2016^23^ |
| GFAP+ | Murine | VSV | mRNA (reporter)/protein |  |  |  | Detje et al. 2015^24^ |
| GFAP+ | Murine | IFNb | mRNA |  | Murine/Human | Ifnar-/- GFAP-Cre+ conditional KO mice, WNV infection, Cytokine/ISG expression | Daniels et al. 2017^25^ |
| GFAP+ |  |  |  |  | Human | ISG (STING/TBK1) staining | Inoue et al. 2021^26^ |
| ASCA2+ |  |  |  |  | Murine | ISG transcripts | Todd et al. 2021^27^ |
| n/a | Human | ZIKV | mRNA |  | Human | ZIKV infection | Das et al. 2022^28^ |

1. Delhaye, S. *et al.* Neurons produce type I interferon during viral encephalitis. *Proc. Natl. Acad. Sci. U. S. A.* (2006) doi:10.1073/pnas.0602460103.

2. Lin, C. C., Wu, Y. J., Heimrich, B. & Schwemmle, M. Absence of a robust innate immune response in rat neurons facilitates persistent infection of Borna disease virus in neuronal tissue. *Cell. Mol. Life Sci.* **70**, 4399–4410 (2013).

3. Baruch, K. *et al.* Aging-induced type I interferon response at the choroid plexus negatively affects brain function. *Science (80-. ).* (2014) doi:10.1126/science.1252945.

4. Reinert, L. S. *et al.* Sensing of HSV-1 by the cGAS-STING pathway in microglia orchestrates antiviral defence in the CNS. *Nat. Commun.* **7**, 1–12 (2016).

5. Khorooshi, R. *et al.* Induction of endogenous Type I interferon within the central nervous system plays a protective role in experimental autoimmune encephalomyelitis. *Acta Neuropathol.* **130**, 107–118 (2015).

6. Bi, Q., Shi, L., Yang, P., Wang, J. & Qin, L. Minocycline attenuates interferon-α-induced impairments in rat fear extinction. *J. Neuroinflammation* **13**, 172 (2016).

7. Wang, J., Campbell, I. L. & Zhang, H. Systemic interferon-α regulates interferon-stimulated genes in the central nervous system. *Mol. Psychiatry* **13**, 293–301 (2008).

8. Rothhammer, V. *et al.* Type i interferons and microbial metabolites of tryptophan modulate astrocyte activity and central nervous system inflammation via the aryl hydrocarbon receptor. *Nat. Med.* **22**, 586–597 (2016).

9. Fares, M. *et al.* Pathological modeling of TBEV infection reveals differential innate immune responses in human neurons and astrocytes that correlate with their susceptibility to infection. *J. Neuroinflammation* **17**, 76 (2020).

10. Lafaille, F. G. *et al.* Impaired intrinsic immunity to HSV-1 in human iPSC-derived TLR3-deficient CNS cells. *Nature* **491**, 769–773 (2012).

11. Li, J. *et al.* Interferon lambda inhibits herpes simplex virus type I infection of human astrocytes and neurons. *Glia* **59**, 58–67 (2011).

12. Roth-Cross, J. K., Bender, S. J. & Weiss, S. R. Murine Coronavirus Mouse Hepatitis Virus Is Recognized by MDA5 and Induces Type I Interferon in Brain Macrophages/Microglia. *J. Virol.* **82**, 9829–9838 (2008).

13. Tarassishin, L., Suh, H.-S. S. & Lee, S. C. Interferon regulatory factor 3 plays an anti-inflammatory role in microglia by activating the PI3K/Akt pathway. *J. Neuroinflammation* **8**, 187 (2011).

14. Kallfass, C. *et al.* Visualizing Production of Beta Interferon by Astrocytes and Microglia in Brain of La Crosse Virus-Infected Mice. *J. Virol.* **86**, 11223–11230 (2012).

15. Welsch, J. C. *et al.* Type I Interferon Receptor Signaling Drives Selective Permissiveness of Astrocytes and Microglia to Measles Virus during Brain Infection. *J. Virol.* **93**, (2019).

16. Hosseini, S. *et al.* Type I Interferon Receptor Signaling in Astrocytes Regulates Hippocampal Synaptic Plasticity and Cognitive Function of the Healthy CNS. *Cell Rep.* **31**, 107666 (2020).

17. Costello, D. A. & Lynch, M. A. Toll-like receptor 3 activation modulates hippocampal network excitability, via glial production of interferon-beta. *Hippocampus* **23**, 696–707 (2013).

18. Thaney, V. E. *et al.* IFNβ Protects Neurons from Damage in a Murine Model of HIV-1 Associated Brain Injury. *Sci. Rep.* **7**, 46514 (2017).

19. Johnson, M. B. *et al.* Retinoic acid inducible gene-I mediated detection of bacterial nucleic acids in human microglial cells. *J. Neuroinflammation* **17**, 1–14 (2020).

20. Barca, O., Devesa-Peleteiro, P., Seoane, M., Señarís, R. M. & Arce, V. M. Bimodal effect of interferon-β on astrocyte proliferation and survival: Importance of nuclear factor-κB. *J. Neuroimmunol.* **226**, 73–80 (2010).

21. Nazmi, A. *et al.* Chronic neurodegeneration induces type I interferon synthesis via STING, shaping microglial phenotype and accelerating disease progression. *Glia* (2019) doi:10.1002/glia.23592.

22. Okada, K. *et al.* Effects of interferon-β on the cytokine production of astrocytes. *J. Neuroimmunol.* **159**, 48–54 (2005).

23. Lindqvist, R. *et al.* Fast type I interferon response protects astrocytes from flavivirus infection and virus-induced cytopathic effects. *J. Neuroinflammation* **13**, 277 (2016).

24. Detje, C. N. *et al.* Upon Intranasal Vesicular Stomatitis Virus Infection, Astrocytes in the Olfactory Bulb Are Important Interferon Beta Producers That Protect from Lethal Encephalitis. *J. Virol.* **89**, 2731–2738 (2015).

25. Daniels, B. P. *et al.* Regional astrocyte IFN signaling restricts pathogenesis during neurotropic viral infection. *J. Clin. Invest.* **127**, 843–856 (2017).

26. Inoue, Y. *et al.* The stimulator of interferon genes (STING) pathway is upregulated in striatal astrocytes of patients with multiple system atrophy. *Neurosci. Lett.* **757**, (2021).

27. Todd, B. P. *et al.* Traumatic brain injury results in unique microglial and astrocyte transcriptomes enriched for type I interferon response. *J. Neuroinflammation 2021 181* **18**, 1–15 (2021).

28. Das, M. *et al.* Astrocyte Control of Zika Infection Is Independent of Interferon Type I and Type III Expression. *Biol. 2022, Vol. 11, Page 143* **11**, 143 (2022).
